# Chemical Composition, In Vitro and In Silico Antioxidant Potential of *Melissa officinalis* subsp. *officinalis* Essential Oil

**DOI:** 10.3390/antiox10071081

**Published:** 2021-07-05

**Authors:** Matilda Rădulescu, Călin Jianu, Alexandra Teodora Lukinich-Gruia, Marius Mioc, Alexandra Mioc, Codruța Șoica, Loredana Gabriela Stana

**Affiliations:** 1 Faculty of Medicine, “Victor Babeş” University of Medicine and Pharmacy, Eftimie Murgu Square 2, RO-300041 Timișoara, Romania; radulescu.matilda@umft.ro (M.R.); stana.loredana@umft.ro (L.G.S.); 2 Faculty of Food Engineering, Banat’s University of Agricultural Sciences and Veterinary Medicine “King Michael I of Romania” from Timisoara, Calea Aradului 119, RO-300645 Timișoara, Romania; 3 OncoGen Centre, County Hospital “Pius Branzeu”, Blvd. Liviu Rebreanu 156, RO-300736 Timișoara, Romania; gruia_alexandra@yahoo.com; 4 Faculty of Pharmacy, “Victor Babeș” University of Medicine and Pharmacy, Eftimie Murgu Square 2, RO-300041 Timișoara, Romania; marius.mioc@umft.ro (M.M.); alexandra.mioc@umft.ro (A.M.); codrutasoica@umft.ro (C.Ș.)

**Keywords:** *Melissa officinalis* subsp. *officinalis*, lemon balm, essential oil, antioxidant activity, molecular docking

## Abstract

The investigation aimed to study the in vitro and in silico antioxidant properties of *Melissa officinalis* subsp. *officinalis* essential oil (MOEO). The chemical composition of MOEO was determined using GC–MS analysis. Among 36 compounds identified in MOEO, the main were beta-cubebene (27.66%), beta-caryophyllene (27.41%), alpha-cadinene (4.72%), caryophyllene oxide (4.09%), and alpha-cadinol (4.07%), respectively. In vitro antioxidant properties of MOEO have been studied in 2,2’-azino-bis(3-ethylbenzothiazoline-6-sulfonic acid) (ABTS) and 2,2-diphenyl-1-picrylhydrazyl (DPPH) free-radical scavenging, and inhibition of *β*-carotene bleaching assays. The half-maximal inhibitory concentration (IC_50_) for the radical scavenging abilities of ABTS and DPPH were 1.225 ± 0.011 μg/mL and 14.015 ± 0.027 μg/mL, respectively, demonstrating good antioxidant activity. Moreover, MOEO exhibited a strong inhibitory effect (94.031 ± 0.082%) in the *β*-carotene bleaching assay by neutralizing hydroperoxides, responsible for the oxidation of highly unsaturated *β*-carotene. Furthermore, molecular docking showed that the MOEO components could exert an in vitro antioxidant activity through xanthine oxidoreductase inhibition. The most active structures are minor MOEO components (approximately 6%), among which the highest affinity for the target protein belongs to carvacrol.

## 1. Introduction

Lipid oxidation represents a significant concern for the food industry because it can occur throughout processing, storage, and distribution, directly affecting food stability, safety, and quality [1]. Furthermore, it can increase oxidative rancidity, loss of essential fatty acids, generation of off odors and off flavors, and toxic compounds, crucial for the foodstuff shelf life [1,2,3]. Consequently, to extend the shelf-life of foodstuffs without any adverse effect on their sensory or nutritional qualities, antioxidants have become an indispensable group of food additives for the food industry, mainly the synthetic ones [3]. They have been reported to act through single or combined mechanisms; particularly, by neutralizing radicals (as radical scavengers), as singlet oxygen quenchers; through synergism with other antioxidants; through complexing of pro-oxidants that catalyze the generation of radicals; and finally, as inhibition of pro-oxidant enzymes that generate radicals (i.e., lipoxygenase, xanthine oxidase, and NADPH oxidase) [4,5,6]. However, due to potential health risks (i.e., carcinogenic and teratogenic effects) [7,8], food consumers have an increasing demand for the development of natural antioxidants, which are generally supposed to be safer [9,10].

The essential oils (EOs) exert various biological activities, prominently antioxidant, antibacterial, and antifungal activities [11,12]. Those properties are mainly associated with EOs’ chemical composition, which is determined by pedoclimatic conditions and plant genotype [13,14,15]. Numerous EOs have been confirmed as natural antioxidants [11,16,17,18] and are recommended as possible replacements of synthetic antioxidants in the food industry. Moreover, the natural extracts’ biological activities can have applicability to the pharmaceutical industry, by inhibiting lipid peroxidative damage associated with pathological disorders, such as aging processes, coronary atherosclerosis, Alzheimer’s disease, and carcinogenesis [19,20]. The antioxidant activity of natural extracts may be due to a combination of multiple factors that commonly result in the reduction in cellular oxidative stress. In addition to the ability of some substances to act as molecules capable of reducing free radicals with destructive oxidative potential, they can act as inducers of enzymes with antioxidant effects, inducers of endogenous antioxidant compound biosynthesis, or inhibitors of enzymes whose metabolic action generates reactive oxygen species (ROS) as a byproduct [21]. Enzymes that can produce ROS are usually involved in metabolic oxidative degradation reactions of endogenous/exogenous compounds. Cytochromes, lipoxygenases, or xanthine oxidoreductase fall into this category. The increased activity of such enzymes can generate a high level of oxidative stress which is usually associated with pathological conditions. Oxidative stress caused by xanthine oxidoreductase hyperreactivity is associated with gout [22], whereas the oxidizing activity of lipoxygenase plays a significant role in oxidative-stress-triggered apoptosis [23]. Other enzymes that produce reactive oxygen species are involved in the regeneration of coenzymes (NADH, NADPH) that regulate the mitochondrial electron transport. Disruption of the physiological activity of such enzymes can cause a mitochondrial imbalance with increasing ROS levels which can have serious implications for cell proliferation, viability, or programmed cell death [24].

*Melissa officinalis* L. (lemon balm), a member of the *Lamiaceae* family, is a perennial subshrub endemic to Europe and Central Asia and extensively cultivated in Romania, Spain, Bulgaria, and Turkey [25]. All three subspecies of *M. officinalis*, subsp. *officinalis*, subsp. *Inodona*, and subsp. *Altissima*, have commercial value, but only subsp. *officinalis* has been extensively cultivated for its characteristic lemon-scented oil [25,26]. *M. officinalis* leaves contain 0.05–0.15% EO in fresh material and 0.1–0.45% EO in dried material, respectively [27]. Due to its digestive and antispasmodic properties, the leaves of *M. officinalis* are utilized in traditional medicine to treat moderate abdominal disorders and biliary dyskinesia [28]. The *M. officinalis* essential oil mainly contains terpenic aldehydes (citral, geranial, neral, and citronellal) and terpenic alcohols (geraniol, linalool, and octen-3-ol-l) [29]. Moreover, EOs and extracts of lemon balm possess antibacterial, antiparasitic, and antiviral activity [18,29,30,31]. Moreover, lemon balm oil and extracts demonstrate good potential for antioxidant activity [18,26,32,33] that recommend them for being used in lipid-containing foods.

The literature review demonstrates a lack of knowledge regarding the antioxidant properties of *Melissa officinalis* L. subsp. *officinalis* essential oil (MOEO) [26,34]. Thus, the purpose of this investigation was to determine the chemical composition of MOEO by GC–MS investigation, the antioxidant activity by 2,2-diphenyl-1-picrylhydrazyl (DPPH), 2,2’-azino-bis(3-ethylbenzothiazoline-6-sulfonic acid) (ABTS), and *β*-carotene/linoleic acid bleaching assays, and to identify other possible protein target-based antioxidant mechanisms of action of the MOEO by means of molecular docking studies.

## 2. Materials and Methods

### 2.1. Raw Material

A sample of *M. officinalis* subsp. *officinalis* (leaves) was collected from Domașnea, Caraș-Severin County (Coordinates: 45°05′ N 22°19′ E) in August 2019. A member of the Faculty of Agronomy authenticated the lemon balm sample. In addition, a voucher specimen of lemon balm (VSNH.BUASTM-88/2) was also deposited for reference purposes in the Herbarium of the Faculty of Agronomy, Banat’s University of Agricultural Sciences and Veterinary Medicine “King Michael I of Romania” in Timișoara. The leaves were dried in a ventilated and sun-sheltered area and stored at 3–5 °C. The MOEO was extracted by steam distillation, as previously described by Jianu et al. [35]. After 3 h of steam distillation, the MOEO was separated by decantation, dried over anhydrous Na_2_SO_4_, and stored at −18 °C in sealed amber vials.

### 2.2. GC–MS Analysis

Volatile compounds contained in the MOEO sample were analyzed with a GC–MS system. First, the sample was diluted 1:1000 in hexane and injected in a GC–MS system in splitless mode. The sample was run on a Br-5MS capillary column, 5% Phenyl-arylene-95% Dimethylpolysiloxane, 30 m length, 0.25 mm internal diameter, 0.25 μm film thickness type (Bruker, Billerica, MA, USA). The oven Gas-Chromatograph (HP6890, Agilent Technologies, Santa Clara, CA, USA) conditions were the following: the analysis started after 3 min of solvent delay, then continued with 6 °C/min in a range of a 50 °C to 300 °C temperature program, and a final hold of 5 min. The Mass Spectrometer (HP5973, Agilent Technologies, Santa Clara, CA, USA) settings were: 230 °C source temperature, 150 °C MS Quad temperature, and 70 eV ionization energy. The helium flow rate was 1 mL/min. The mass range of compounds was scanned between 50 to 550 amu. Identification of the MOEO compounds was based on computer matching with the mass spectra from the NIST0.2 library (USA National Institute of Science and Technology software, NIST, Gaithersburg, MD, USA) and retention indices (RIs) compared with the literature Adams indices [36].

### 2.3. DPPH• Free-Radical Scavenging Activity

Antioxidant scavenging activity was investigated by 2,2-diphenyl-1-picrylhydrazyl (DPPH) free radical-scavenging assay previously described by Jianu et al. [37]. A total of 10 μL from a DPPH stock solution of 1 mg/mL was mixed with 100 μL of serial dilutions between 1.5 to 0.093 mg/mL of MOEO. All samples were put in triplicate in a 96 well plate and stored at 25 °C for 30 min in the dark. The absorbance readings were performed at a 515 nm DPPH wavelength and were registered as A_1_, using Tecan i-control, 1.10.4.0 infinite 200Pro spectrophotometer (Tecan Group Ltd., Männedorf, Switzerland). The same protocol was applied to the positive control samples, represented by the BHA and ascorbic acid solutions; the blank samples’, represented by a solution without MOEO, absorbance registered as A_0_. Results expressed the DPPH free radical inhibition percent and were calculated with the equation: I% = (A_0_ − A_1_)/A_0_ 100. IC_50_ (μg/mL) was determined using the free BioDataFit 1.02 software (Chang Broscience Inc., Castro Valley, CA, USA). Experiments were conducted in triplicate.

### 2.4. ABTS•^+^ Free-Radical Scavenging Activity

The scavenging capacity to the ABTS radical of the MOEO was investigated, as described by Re et al. [38] with some modifications [39]. First, a fresh ABTS^+^ solution was obtained in a mixture of 7 mM of ABTS at pH 7.4 (5 mM NaH_2_PO_4_, 5 mM Na_2_HPO_4_, and 154 mM NaCl) and 2.5 mM potassium persulfate (final concentration), followed by storage at 25 °C up to 16 h before use. The absorbance of the ABTS^+^ solution was adjusted to 0.700 ± 0.038 at 734 nm before use [40]. Next, aliquots (100 μL) of various concentrations (ranging from 1.5 to 0.093 mg/mL) of the MOEO in methanol and the reference antioxidants (BHA and ascorbic acid) were added to the ABTS^+^ solution (1 mL) and mixed vigorously. The absorbances were measured at 734 nm after 10 min of incubation (at 25 °C in the dark). IC_50_ (μg/L) was determined using the free BioDataFit 1.02 software (Chang Broscience Inc., Castro Valley, CA, USA). Experiments were conducted in triplicate.

### 2.5. β-Carotene/Linoleic Acid Bleaching Assay

The investigation was conducted using the method reported by Jianu et al. [41]. First, a stock solution was obtained by dissolving 0.5 mg *β*-carotene in 1 mL of chloroform, then mixed with 200 mg Tween 40 and 25 μL linoleic acid. Next, the chloroform was removed under vacuum at 40 °C for 5 min using a rotary evaporator (Heidolph, Schwabach, Germany). Subsequently, 100 mL of 3% hydrogen peroxide aqueous solution were added to the residue and mixed vigorously for 2–3 min until obtaining an emulsion. Finally, aliquots (2.5 mL) of the emulsion were transferred to the test tubes containing MOEO (350 μL); BHA was used as the reference antioxidant. All test tubes were incubated at 25 °C up to 48 h, before measuring the absorbances at 490 nm. Experiments were conducted in triplicate.

### 2.6. In Silico Prediction of Bioactivity and Molecular Docking Studies

Crystalographic 3D protein structures were obtained from the RCSB Protein Data Bank [42] (Table 1).

Protein structures were prepared as suitable docking targets using Autodock Tools (version 1.5.6). Water molecules, metal atoms, the co-crystalized ligands, and other non-covalent bonded molecules were removed from the protein structures. Gesteiger charges were added, after which the target file was saved as a suitable pdbqt format. When saved, the software automatically adds polar hydrogen/merges nonpolar hydrogens to the structure. Ligand structures corresponding to all 36 *M. officinalis* identified compounds were drawn as 2D mol files (Biovia Draw, Dassault Systems Biovia, San Diego, CA, USA) and were subsequently converted into 3D optimized structures using PyRx’s embedded Open Babel function, using the universal force field (uff). Rigid molecular docking was performed using PyRx v0.8 (The Scripps Research Institute, La Jolla, CA, USA), employing Autodock Vina’s embedded scoring function [43]. Our proposed docking method was validated by re-docking the native ligands into their original binding pockets. The predicted docked conformation was superimposed onto the experimental binding conformation, and root means square deviation values were calculated for the two poses. Docking studies were performed only for protein structures where the RMSD values between the native ligand’s docked and experimental pose did not exceed a 2 Å threshold. The grid box corresponding to the docking search space was adjusted to fit the active binding site best. Grid box coordinates are listed in Table 1. Obtained results for the docked ligand structures were recorded as ΔG binding energy values (kcal/mol). Protein-ligand binding interactions were examined using Accelerys Discovery Studio 4.1 (Dassault Systems Biovia, San Diego, CA, USA).

### 2.7. Statistical Analysis

IBM SPSS 25.0 software (SPSS Inc., Wacker Drive, Chicago, IL, USA) was used to calculate the mean and standard deviation of three independent tests involving triplicate analyses for each sample. A post-hoc test (Tukey) was applied to test for significant differences between the mean values obtained from antioxidant activity measurements (at the 5% level).

## 3. Results and Discussion

### 3.1. MOEO Chemical Composition

A pale-yellow color oil with a lemon-like odor was isolated by steam distillation from *M. officinalis* leaves with a 0.41% yield. The determined yield revealed that the plant sample from western Romania is rich in essential oil. Moreover, the results match the scientific literature’s values that report yields ranged between 0.01 and 0.45% (dry material) [44]. Higher yields have been recorded for *M. officinalis* from Brazil (0.97%) [45], Iran (1%) [46], and Spain (0.8%) [47]. According to Kittler et al. [44], the lemon balm EO content is strongly related to the biotic and abiotic conditions, different harvesting years, and genetic makeup of the genotypes.

The GC–MS analysis identified 36 components, representing 98.79% of the total contents of the MOEO (Table 2). The main constituents are beta-cubebene (27.66%), beta-caryophyllene (27.41%), alpha-cadinene (4.72%), caryophyllene oxide (4.09%), and alpha-cadinol (4.07%). A high content of sesquiterpenoids, such as beta-cubebene (15.41%), beta-caryophyllene (14.24%), alpha-cadinol (7.19%), has also been reported in a MOEO from Turkey [48]. According to the scientific data, beta-cubebene is a chemical compound commonly found in lower amounts in subsp. *officinalis* [15,28]. Only for the subsp. *altissima* was there previously recorded a higher amount of beta-cubebene (39%) [49]. However, caryophyllene, the second major compound of the analyzed oil, has been recorded in large amounts in subsp. *officinalis* from Sardinia (20–39%) [50] and Germany (1.17–18.64%) [44]. Another peculiarity of the analyzed oil is the low content of alpha-citral (2.06%), beta-citral (1.15%), and citronellal (0.27%), compared with other subsp. *officinalis*. These oxygenated monoterpenes are present in large amounts in subsp. *officinalis* EOs [15,18,28] and are responsible for their lemon-like aroma [49]. This phytochemical polymorphism is significantly determined by genetic factors [44,51] and also influenced by ontogenetic [52] and environmental variations [53].

### 3.2. Assessment of Antioxidant Activity

The antioxidant activity of MOEO was evaluated by three in vitro tests, DPPH, ABTS, and *β*-carotene/linoleic acid bleaching assays. Results are displayed as mean ± SD of triplicate tests in Table 3. In the DPPH assay, the MOEO’s ability to act as the donor for hydrogen atoms or electrons in the transformation of DPPH^•^ into its reduced form DPPH-H was measured spectrophotometrically. The MOEO was able to reduce the stable radical DPPH to the yellow-colored DPPH-H, reaching a 50% reduction with a IC_50_ of 14.015 ± 0.027 μg/mL. A comparison of the DPPH scavenging activity of MOEO to those expressed by BHA pointed out very similar IC_50_ values (11.006 ± 0.011 μg/mL) with no significant difference (*p* > 0.05) observed by the Tukey test. Furthermore, the scavenging ability of the MOEO was significantly (*p* < 0.05) higher than that of ascorbic acid (618.117 ± 0.174 μg/mL). These results are comparable with previous studies that report a strong DPPH^•^ free radical scavenging capacity for EO [18,54,55,56] and extract isolated from *M. officinalis* [33,54,55,57].

The ABTS coloring method is an excellent method for determining the antioxidant activity of a broad diversity of substances, such as hydrogen-donating antioxidants or scavengers of aqueous phase radicals and chain-breaking antioxidants or scavengers of lipid peroxyl radicals [38]. In the ABTS radical scavenging method, MOEO showed a strong antioxidant activity with a IC_50_ of 1.225 ± 0.011 μg/mL (Table 3), which was significantly (*p* < 0.05) more pronounced than that of ascorbic acid, IC_50_ value 29.434 ± 0.081 μg/mL. However, BHA has a better ability to scavenge ABTS^•−^ radicals, displaying a IC_50_ value of 0.902 ± 0.003 μg/mL, with no significant difference (*p* > 0.05) observed. The obtained results appear to be better than the findings of Dastmalchi et al. [58] and Ben et al. [59] for ethanolic extracts and Ehsani et al. [46] for EO of *M. officinalis*.

The *β*-carotene/linoleic acid bleaching assay determines the antioxidants’ ability to protect target molecules exposed to a free radical source and antioxidants’ capacity to inhibit or delay lipid oxidation [60]. The assay employs a model lipid substrate, conceded to be a good model for membrane-based lipid peroxidation [54]. The antioxidant activity of MOEO expressed as relative antioxidant activity (RAA%) was calculated with the equation: RAA = A_MOEO_/A_standard_ (A_standard_ is the absorption of BHA, the positive control used, and A_MOEO_ is the absorption of MOEO). MOEO exhibited strong antioxidant activity (94.031 ± 0.082%) in the *β*-carotene-linoleic acid test, but lower than that of BHA (100%) (Table 3). No significant differences (*p* > 0.05) in their efficacy were observed. Similar results were recorded for extracts obtained from subsp. *officinalis* and subsp. *altissima* [26].

The activity of plant-origin natural extracts is often evaluated for their proposed antioxidant activity using established methods such as those used in our current study. The ability of a natural compound or extracts to scavenge free radicals such as DPPH∙ or ABTS∙ reflects its ability to act similarly in the presence of ROS at the cellular/mitochondrial levels. Numerous studies have shown a clear correlation between the ability of an extract to scavenge free radicals assessed by the DPPH or ABTS method and the ability of the same product to decrease ROS production in vitro. Wettasinghe et al. assessed the ROS and DPPH^•^ scavenging capacity of a borage and evening primrose crude extracts and several standardized fractions [61]. Their results clearly showed that the most active tested extracts and fractions inhibited DPPH^•^ and ROS formations in a dose-dependent manner. A more recent study evaluated the antioxidant activity of several *Solanum sisymbriifolium* extracts showing that the tested products also demonstrate the ability to scavenge DPPH^•^ and ABTS^•^ and, at the same time, reduce ROS production in a dose-dependent manner [62]. Although these correlations may be difficult to consider due to the complex composition of a plant extract, they are also reported in cases when the antioxidant activity of a single chemical compound is determined. Bai et al. showed that dimethylglycine sodium salt exerted its free radical scavenging capacity against DPPH, ABTS, and H_2_O_2_ and reduced ROS production at the same time [63]. Considering our obtained results, we can conclude that the MOEO has in vitro antioxidant potential through the ability to scavenge free radicals such as cellular/mitochondrial level ROS.

### 3.3. In Silico Prediction of a Protein Target-Based Antioxidant Mechanism by Molecular Docking Analysis

Terpenoids are secondary metabolites in plants and are often used as natural starting compounds in drug development. Their biological properties are due to their ability to target or regulate the activity of key enzymes involved in proliferation, inflammation, or oxidative stress [64]. Such possible biological effects can be predicted through state-of-the-art computational techniques with continuously increasing prediction capacity. These computational methods are extensively used in different stages of modern-day drug discovery research, aiding scientists in their ongoing quest for developing potent therapeutical active compounds. Molecular docking is a useful technique that can aid in an advanced understanding of plausible action mechanisms exhibited by in vitro biological active molecules.

Herein we used molecular docking to identify a supplementary possible protein-targeted mechanism of action correlated with the potential in vitro antioxidant effect of the terpene-rich MOEO.

For the present study, we chose to investigate, using an in silico-based approach, the potential of the MOEO components to act as inhibitors against available target proteins involved in intracellular antioxidant mechanisms or reactive oxygen species (ROS) generation. For this purpose, lipoxygenase, CYP2C9, NADPH-oxidase, xanthine oxidase, and type II—NADH dehydrogenase were used as protein targets.

Obtained docking scores are listed as a three-colored scheme (red-yellow-green) heat map table that can easily show a clear tendency of a set of compounds to act as potential inhibitors for a certain protein. For each protein target, the color range was set from red (as the energy value corresponding to the native ligand) to green, spanning a 5 kcal/mol interval (Table 4). This approach is applicable especially for sets of compounds that share a high structural similarity. In our case, out of the 37 EO-tested components, the vast majority is represented by monoterpenes/monoterpene derivatives.

Our results show that the EO compounds have a clear tendency to act as xanthine oxidoreductase (PDB ID: 3NRZ) inhibitors. Out of the 37 tested compounds, 7 structures gave docking scores higher or equal to that of the native ligand hypoxanthine. Xanthine oxidoreductase is the enzyme that catalyzes the oxidation of hypoxanthine to xanthine and the subsequent transformation of xanthine to uric acid. In addition to this biological role, mammalian xanthine oxidoreductase is a physiological source of ROS, such as superoxide ions or hydrogen peroxide, which can trigger the activation of various pathways [65]. Therefore, the inhibition of this particular enzyme could induce a significant in vitro antioxidative effect. According to the obtained docking scores, among the most active compounds are various structures, including monoterpenes (carvacrol, −7.2 kcal/mol), monoterpenoid esters (methyl geranate, −7 kcal/mol), or sesquiterpenes (alpha-farnesene, −7.1 kcal/mol). Xanthine oxidoreductase inhibitors are usually researched for their potential effect in reducing the oxidative stress present in gout. On the same note, a similar study aimed to determine the inhibitory activity of some commercially available mono- and sesquiterpenes [66]. Published data showed that the assessed compounds showed superior in silico inhibitory activity of xanthine oxidoreductase as compared to allopurinol. Compounds such as beta-caryophyllene (a MOEO constituent) or alpha-terpinene (an isomer of the corresponding MOEO gamma-terpinene) gave similar docking scores as the ones reported in this study. Moreover, the authors determined that these compounds also show an in vitro xanthine oxidoreductase inhibitory activity. The seven most active compounds also account for approximately 6% of the total EO composition. This could mean that even if a biological antioxidant effect could well be correlated to the synergistic activity of most compounds, the highest in vitro antioxidant effect related to xanthine oxidoreductase inhibition is actually attributed to minor-occurring molecules. Our results are consistent with previous in silico studies reported by our research group, where a monoterpene-rich *Mentha smithiana* EO gave similar results in terms of a proposed in vitro antioxidant activity by targeting xanthine oxidoreductase [67].

**Figure 1:**
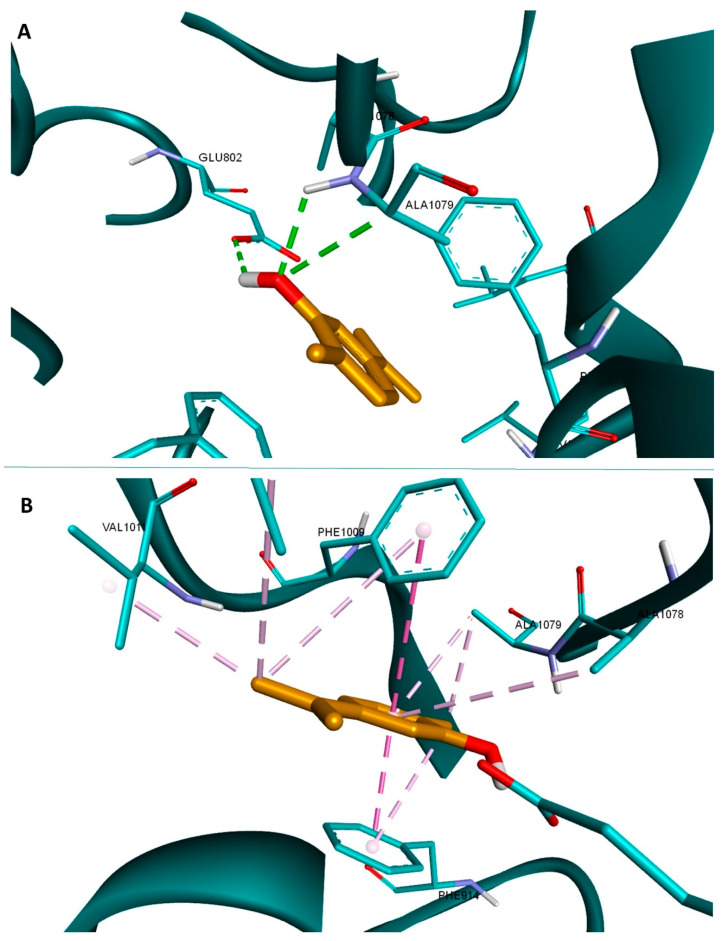
Structure of xanthine oxidoreductase (3NRZ) in complex with carvacrol (orange); hydrogen bond interactions are depicted as green dotted lines (**A**), and hydrophobic interactions as purple dotted lines (**B**); interacting amino acids are shown as light blue sticks.

The most active docked compound according to the obtained scores was carvacrol. Our predicted results are in line with a recent study published by Rezaienasab et al. according to which carvacrol inhibits xanthine oxidoreductase in a dose-dependent manner, and its antioxidant activity is related to the decrease om ROS production due to xanthine oxidoreductase inhibition [68]. Binding interactions analysis of carvacrol reveals the formation of three hydrogen bonds, one with Glu802 and the other two with Ala1079 (Figure 1A), similar to the binding pattern of the native ligand. The structure is also very well stabilized in the binding pocket through multiple hydrophobic interactions (Figure 1B). Given the existing above mentioned biological literature data that validate our docking method, we can conclude that MOEO is a strong antioxidant product that could exert its antioxidant activity not only by radical scavenging but also by targeted inhibition of xanthine oxidoreductase.

## 4. Conclusions

The study showed that the analyzed MOEO is rich in beta-cubebene (27.66%) and beta-caryophyllene (27.41%). The antioxidative data recorded demonstrate that the lemon balm essential oil possesses a significantly (*p* < 0.05) higher scavenging ability than ascorbic acid. Moreover, MOEO antioxidant abilities in DPPH, ABTS, and *β*-carotene/linoleic acid bleaching assays were comparable with those of BHA; even no significant differences (*p* > 0.05) in their efficacy were observed. These results suggest that MOEO can act as a promising antioxidant product by free radical scavenging. Molecular docking showed that the MOEO components could also exert a protein-targeted in vitro antioxidant activity through xanthine oxidoreductase inhibition. From the tested compounds, the most active seven structures (registered equal or lower binding affinities compared to the native ligand) are minor oil components (approximately 6%), among which the highest affinity for the target protein belongs to carvacrol. In conclusion, MOEO can be a potential new source of natural antioxidants in the pharmaceutical and food industries.

## Figures and Tables

**Table 1:** Molecular docking parameters and protein targets.

Protein	PDB ID	Grid Box Center Coordinates	Grid Box Size	Conformers Generated per Ligand
**Lipoxygenase**	1N8Q	center_x = 22.4550	size_x = 12.4283	8
		center_y = 1.2929	size_y = 10.6802	
		center_z = 20.3621	size_z = 12.1421	
**CYP2C9**	1OG5	center_x = −19.8236	size_x = 12.3877	8
		center_y = 86.6869	size_y = 11.6533	
		center_z = 38.2757	size_z = 11.6542	
**NADPH-oxidase**	2CDU	center_x = 18.9974	size_x = 14.0073	8
		center_y = −5.7774	size_y = 14.9976	
		center_z = −1.8087	size_z = 18.7956	
**Xanthine oxidase**	3NRZ	center_x = 37.4800	size_x = 7.3289	8
		center_y = 19.3054	size_y = 10.3411	
		center_z = 18.1518	size_z = 9.1241	

**Table 2:** Chemical composition of *M. officinalis* subsp. *officinalis* essential oil analyzed by GC–MS.

No	Compounds	RI ^1^	%
1.	Hydroperoxide, 1-ethylbutyl	925	0.11
2.	Hydroperoxide, 1-methylpentyl	934	0.08
3.	*p*-Cymene	1005	0.07
4.	beta-*trans*-Ocimene	1017	0.09
5.	beta-*cis*-Ocimene	1029	0.51
6.	gama-Terpinene	1042	0.09
7.	Nonanal	1092	0.17
8.	(*R*)-(+)-Citronellal	1145	0.27
9.	Decanal	1206	0.11
10.	Octyl acetate	1211	0.08
11.	beta-Citral	1241	1.15
12.	(*S*)-(−)-Citronellic acid, methyl ester	1264	0.66
13.	alpha-Citral	1275	2.06
14.	Carvacrol	1309	0.18
15.	Methyl geranate	1333	0.19
16.	*p*-Menthane-3,8-diol	1352	2.14
17.	alpha-Copaene	1394	2.78
18.	beta-Bourbonene	1402	1.16
19.	beta-Elemene	1408	2.73
20.	beta-Caryophyllene	1442	27.41
21.	alpha-Cubebene	1450	0.41
22.	alpha-Caryophyllene	1476	3.37
23.	Alloaromadendrene	1481	0.87
24.	beta-Cubebene	1504	27.66
25.	(*Z,E*)-alpha-Farnesene	1512	1.37
26.	alpha-Muurolene	1520	0.96
27.	alpha-Farnesene	1526	0.71
28.	gamma-Cadinene	1534	1.36
29.	alpha-Cadinene	1540	4.72
30.	Germacrene D-4-ol	1596	1.96
31.	Caryophyllene oxide	1601	4.09
32.	alpha-Cadinol	1669	4.07
33.	Isoaromadendrene epoxide	1819	0.98
34.	Platambin	1849	2.13
35.	Murolan-3,9(11)-diene-10-peroxy	1884	1.18
36.	Aromadendrene oxide	1891	0.92
		Total:	98.79

^1^ The retention index (RI) was calculated upon a calibration curve obtained by injecting in the same conditions as the samples of a C_8_-C_20_ alkane standard mixture.

**Table 3:** Antioxidant activities of *M. officinalis* subsp. *officinalis* essential oil.

Samples Tested	Parameters		
	DPPH, IC_50_ (μg/mL)	ABTS, IC_50_ (μg/mL)	β-Carotene/Linoleic Acid, (% Inhibition Rate)
MOEO	14.015 ± 0.027	1.225 ± 0.011	94.031 ± 0.082
BHA ^1^	11.006 ± 0.011	0.902 ± 0.003	100
Ascorbic acid	618.117 ± 0.174	29.434 ± 0.081	N.T.

^1^ BHA—butylated hydroxyanisole; ±: standard deviation; N.T.—not tested.

**Table 4:** Heat map of recorded docking scores (binding free energy—kcal/mol) of the *M*. *officinalis* subsp. *officinalis* essential oil components.

	Protein PBD ID	1N8Q	1OG5	2CDU	3NRZ
Ligand		Binding Free Energy ∆G (kcal/mol) ^1^			
Native co-crystalized ligand		−5.8	−9.8	−9.3	−6.7
Hydroperoxide, 1-ethylbutyl		−4.6	−6.4	−6.1	−5.7
Hydroperoxide, 1-methylpentyl		−3.3	−5.8	−6	−5.8
*p*-Cymene		−5.1	−4.8	−4.4	−6.9
beta-*trans*-Ocimene		−5	−5.1	−5.1	−6.3
beta-*cis*-Ocimene		−6	−6.7	−6	−6.2
gamma-Terpinene		−5.6	−6	−5.9	−6.8
Nonanal		−6.5	−5.7	−5.6	−6.4
(*R*)-(+)-Citronellal		−0.6	−7.3	−6.8	−6.4
Decanal		−5.3	−5.5	−4.9	−6.5
Octyl acetate		−5.1	−5.9	−5.5	−6.5
beta-Citral (Neral)		−4.4	−7.4	−6.8	−6.3
(*S*)-(−)-Citronellic acid, methyl ester		−2.8	−5.7	−5.3	−6.5
alpha-Citral (Geranial)		−3.8	−7.6	−7.3	−6.4
Carvacrol		−3.9	−5.7	−5.6	−7.2
Methyl geranate		−0.4	−7.7	−7.2	−7
*p*-Menthane-3,8-diol		−5.8	−6.3	−6.1	−6.7
alpha-Copaene		−4	−5.9	−5.9	−6.1
beta-Bourbonene		−5.3	−6.5	−6.2	−6.2
beta-Elemene		−3.5	−6.7	−6.4	−6.4
beta-Caryophyllene		−4.9	−6.4	−6.1	−5.2
alpha-Cubebene		−5.9	−6.1	−6	−5.2
alpha-Caryophyllene		−4.4	−6.4	−5.8	−5.1
Alloaromadendrene		−5.3	−6.3	−5.9	−5.1
beta-Cubebene		−4.7	−5.8	−5.7	−5.1
(*Z,E*)-alpha-Farnesene		−3.3	−5.5	−5.9	−7.1
alpha-Muurolene		−4.4	−5.6	−5.7	−5.9
alpha-Farnesene		−3.2	−6.8	−6	−7
gamma-Cadinene		−5.2	−6	−6.3	−6.7
alpha-Cadinene		−5.6	−6.3	−5.7	−6.5
Germacrene D-4-ol		−4.8	−5.5	−5	−5
Caryophyllene oxide		−4.6	−6.3	−5.6	−4.5
alpha-Cadinol		−4.9	−4.9	−4.7	−5
Isoaromadendrene epoxide		−6	−6.2	−5.7	−4.5
Platambin		−5.5	−6.3	−5.7	−3.1
Murolan-3,9(11)-diene-10-peroxy		−4	−6.1	−5.8	−5.2
Aromadendrene oxide		−1.2	−7.9	−7.1	−4.2

^1.^ Color scale varies, for each set (column), from red (native ligand recorded ∆G), through yellow (mid-point), to green (native ligand recorded ∆G +5 kcal/mol).

## Data Availability

The data presented in this study are available on request from the corresponding author.
